# Understanding the relationship between adolescents with tuberculosis and health services: an indepth qualitative study from Cape Town

**DOI:** 10.1136/bmjopen-2024-094295

**Published:** 2025-05-24

**Authors:** Dillon Timothy Wademan, Graeme Hoddinott, Zara Kavalieratos, Mfundo Mlomzale, Arlene J Marthinus, Lucia N Jola, Stephanie Jacobs, Khanyisa Mcimeli, James Seddon

**Affiliations:** 1 Desmond Tutu TB Centre, Department of Paediatrics and Child Health, Stellenbosch University Faculty of Medicine and Health Sciences, Cape Town, Western Cape, South Africa; 2 Department of Global and Public Health, The University of Sydney, Sydney, New South Wales, Australia; 3 Department of Paediatric Infectious Diseases, Imperial College London, London, UK

**Keywords:** Tuberculosis, Adolescents, QUALITATIVE RESEARCH

## Abstract

**Abstract:**

**Introduction:**

Adolescents’ experiences (10–19 years-old) with tuberculosis (TB) remain poorly understood. Descriptions of adolescent TB experiences, particularly how they interact with the health system, are scarce. We aimed to understand adolescents’ experiences of TB health services in the Western Cape, South Africa. We focused on how TB services were aided or hindered through interactions with healthcare providers and health system processes.

**Methods:**

Teen TB, an observational study in Cape Town, enrolled 50 newly diagnosed adolescents with multidrug-resistant and drug-susceptible TB. A subset of 20 was selected for serial qualitative data collection, with 19 completing all tasks between December 2020 and September 2021. 52 interviews were conducted and thematically analysed using a case descriptive process for experiences across the TB care cascade.

**Findings:**

Adolescents criticised the difficulties and delays they encountered in obtaining an accurate TB diagnosis. Initial misdiagnoses and delayed TB diagnoses were reported, despite seeking help from multiple healthcare providers at different facilities. Adolescents questioned whether the financial, social and emotional costs of TB care outweighed the costs of delaying treatment initiation and adherence. Adolescents reported that the treatment regimen, adherence support processes and interactions with the health system posed significant challenges to maintaining adherence. Encouragingly, however, most adolescents reported being well treated and cared for by health workers.

**Conclusion:**

Our study shows that adolescents experience challenges throughout their TB treatment journeys. More adolescent-focused research is needed to tailor treatment and healthcare processes to their needs.

**STRENGTHS AND LIMITATIONS OF THIS STUDY**

- Care provided within the study setting may not reflect the unmet care needs of adolescents in routine care settings.
- Data collection occurred during the height of the COVID-19 pandemic, which negatively impacted the tuberculosis (TB) healthcare services.
- Transferability of the findings is limited by the small sample size recruited from a single, clinical site.
- The study included adolescents across a broad age range, diverse ethnicities and various forms of TB (with and without HIV co-infection).
- Longitudinal data collection, coupled with iterative data refinement, ensured analytic rigour.

## Introduction

 An estimated 750 000 adolescents (defined as individuals from 10 years to <20 years) develop tuberculosis (TB) disease each year globally, almost all in low-and middle-income countries with a high burden of TB.^1 2^ The risk of progression from *Mycobacterium tuberculosis* (*Mtb*) infection to TB disease rises rapidly in adolescence.^3^ This may be related to the impact of puberty and associated hormonal changes on immunological responses to *Mtb* or due to co-infections (especially HIV), poor nutrition and substance use (alcohol, smoking and illicit drugs) impacting on TB pathogenesis.^4^ Adolescents commonly develop cavitary forms of pulmonary TB disease—like adults.^5^ Adolescents also typically have multiple and broad social networks, frequently present late to care and experience high rates of loss to follow-up, all of which propagate the epidemic.^6^,^8^ TB has been identified as a key research priority for adolescent health in low- and middle-income countries, and adolescent TB is a crucial component of the revised WHO Roadmap for ending TB.^9 10^ Despite the pressing need to better understand adolescent TB, few studies have examined this topic. Unless the experiences of adolescents are better characterised and their needs are addressed, it will be impossible to meet the ambitious targets set by the WHO End TB Strategy.^11 12^

One way of organising an analysis of the interactions between adolescents and TB care services is through use of the TB care cascade.^13 14^ Losses at every step along the TB cascade persist for adolescents, yet little is known about factors contributing to these drop-offs at each stage or about adolescents’ experiences of their TB diagnosis, treatment initiation and ongoing care.^15^ Adolescents have steeper losses and poorer outcomes at each step in the care cascade compared with adults and younger children.^1 6 16^

Multiple social and structural conditions intersect during adolescence that impact on adolescents’ risk of TB disease, disease progression and health outcomes. Adolescents frequently attend overcrowded and understaffed primary healthcare (PHC) facilities, offering ‘unfriendly’ healthcare services.^17 18^ Adolescents’ experiences with health systems impact their health outcomes.^19 20^ Fear of discrimination and disclosure and lack of social and economic support have been shown to complicate healthcare and treatment services for adolescents.^21 22^ As adolescents age into early adulthood, additional factors contribute to the difficulties in engagement with healthcare services, including high unemployment and geographical mobility.^23 24^ However, adolescents with TB are not a homogeneous group. Adolescents with TB have different demographic, clinical and socioeconomic characteristics, and these impact their TB risk, treatment outcomes and the long-term impact of TB on their lives in varying ways. Emerging autonomy, changing peer relationships and social and educational needs are all impacted in different ways dependent on stage of adolescence and gender.^25^

Drug resistance in the infecting *Mtb* isolate has a profound impact on how an individual experiences TB, and while TB incidence has decreased over the past decade, multidrug-resistant (MDR)-TB incidence has increased.^26^,^28^ MDR-TB is defined as disease caused by *Mtb* resistant to isoniazid and rifampicin. HIV also increases the risk of TB, can make the diagnosis more challenging, can complicate treatment and can affect health outcomes.^29 30^

A key element of improving outcomes for adolescents with TB is to better understand their interaction with health services—to mitigate these biopsychosocial effects and optimise their care experiences. We aimed to understand the experiences of adolescents with TB as they interact with health services. Specifically, our objectives were to: (1) describe adolescents’ experiences of their TB diagnosis and treatment pathways and (2) identify health service delivery issues that are linked to both positive and negative experiences of adolescents along their care journeys.

## Methods

### Setting

Cape Town is the second most populous metropolitan area in South Africa with around~5 million residents. TB services in Cape Town are delivered primarily as outpatient care through government PHC facilities with referral to general or TB-specialised hospitals, where deemed clinically necessary. Between December 2021 and December 2022, there were a total (all ages) of 21 124 diagnosed TB cases within the City of Cape Town Metro with 2439 TB-associated deaths and 1275 reported cases of drug-resistant TB (defined as known resistance to any TB antibiotic).^31^ Over the study period, diagnosis at primary care was primarily through Xpert MTB/RIF on expectorated sputum samples, with treatment initiation and ongoing care at that clinic for both drug-susceptible TB (DS-TB) and DR-TB. At the time of the study, the standard of care treatment for adolescents with DS-TB in the Western Cape comprised a 2-month intensive phase with isoniazid, rifampicin, pyrazinamide and ethambutol, followed by a 4-month continuation phase with isoniazid and rifampicin. Adolescents with MDR-TB received injectable-free treatment, with durations ranging from 9 months to 20 months, depending on their drug resistance profile.

### Teen TB

The *‘Understanding the biology, morbidity and social contexts of adolescent tuberculosis (Teen TB)’* study, which took place in February 2020–2022, was an observational prospective cohort study among adolescents with newly diagnosed microbiologically confirmed DS-TB or MDR-TB disease in Cape Town, Western Cape, South Africa.^32^ In Teen TB, 50 adolescents with TB and 50 adolescents without TB but who were exposed to TB in their household (controls) were recruited. Adolescents were included in the TB arm of Teen TB if they were adolescents (10–19 years old) with newly diagnosed pulmonary TB bacteriologically confirmed on sputum (Xpert MTB/RIF positive or culture-positive), with or without HIV co-infection, regardless of drug resistance testing profile and who were within the first 14 days of treatment, a cut-off that ensured we only recruited adolescents soon after TB diagnosis and treatment initiation. HIV testing was performed if the individual did not have a negative HIV test in the preceding 6 months, by a trained HIV counsellor, following informed consent and with pretest and post-test counselling. This manuscript reports on the qualitative substudy conducted within the Teen TB study.

### Patient and public involvement

The development of the Teen TB protocol, the ethics submission and the strategy for implementation of the study were undertaken in close collaboration with the Desmond Tutu TB Centre’s Community Advisory Board.

### Design and sampling

We conducted a qualitative study among a subsample of the Teen TB participants. 20 participants with TB disease, sampled purposively for diversity in gender, age and drug resistance type, were invited to the qualitative study (table 1). 19 participants completed all study activities; one participant was lost to attrition. Participants were recruited during Teen TB clinical visits and consented separately for participation in the qualitative substudy. We purposively enrolled adolescents with TB by diversity in age, gender and drug susceptibility to gain a range of experiences, until reaching saturation for meaning.^33^

**Table 1:** Participant information for those included in the qualitative study

	Characteristics	Male	Female	All
Age	Median age (IQR)	17.5 (5.5)	16 (2.5)	17 (4.25)
	Age range (years)	12–19	13–19	12–19
	HIV positivity	2	2	4
	Prior TB episode	2	2	4
Drug susceptibility	Drug-susceptible	7	4	11
	Drug-resistant	3	6	9

TB, tuberculosis.

### Data collection processes

The qualitative data comprised 2–3 semistructured, indepth interviews (~30–90 min) with each participant, over a period of about 6 months, between December 2021 and September 2022, while they were on TB treatment. Interviews were conducted at the clinical visits, as part of the Teen TB study. Adolescents chose who attended, controlling their privacy. Participatory research activities (including kinship and body mapping) were used during each interview to elicit discussions about adolescents’ experiences of TB disease, treatment and health services, familial/social contexts, sexual experiences, HIV, substance use and the impact of TB on education.^34^ Each participant was interviewed by a postgraduate research assistant (SJ, KM) with whom they shared a language fluency (English, Xhosa or Afrikaans). We believe the facts that both research assistants were young, well-educated women, who spoke participants’ mother tongue and shared their ethnic background, likely facilitated building rapport with participants and contributed to their willingness to share their experiences. The research assistants had prior experience collecting data with young people and received study-specific training to avoid any potential barriers to communicating with adolescent participants. In total, 52 semistructured interviews were conducted with 19 participants. The interviews were audio recorded and photographs taken of participatory activities, and detailed case descriptions were written after each interaction with participants, which were iteratively refined with senior sociobehavioural scientists (DTW, GH).

### Data analysis

Analysis comprised three phases.^35^ The first phase entailed developing iteratively refined case descriptions of each participant’s overall experiences, across the interviews and data collection activities were collated into a single case file per participant (led by SJ and KM with support from DTW and GH). The second phase involved two graduate sociobehavioural scientists (ZK, LNJ) reading and re-reading all case files and listening to the audio recordings of participant interviews to further supplement these case files with relevant quotes. Four parameters of adolescents’ experiences of TB and health service interactions, loosely based on the TB care cascade, underpinned the deductive thematic analysis, namely, (1) adolescents’ diagnostic and treatment pathways, (2) adolescents’ experiences of diagnostic tools and processes, (3) treatment adherence challenges and (4) perceptions of interactions with healthcare systems and healthcare providers. Phase three entailed ZK, LNJ, MM and DTW further analysing the refined case files following a deductive thematic analysis approach.

## Findings

### Prediagnosis and treatment pathways

Participants across categories (gender, language and drug susceptibility pattern) reported similar decisions and experiences of symptoms and healthcare seeking practices (table 2). Participants were hesitant to seek care—even participants with prior TB experience (either personal or within their household)—due to financial, social and emotional costs of seeking care. Downplaying the severity of symptoms, prioritising schoolwork or family responsibilities, fear of being diagnosed with TB (again) and fear of COVID-19 were among the reasons adolescents provided for avoiding or delaying seeking care. Another factor delaying a TB diagnosis was adolescents being misdiagnosed. Adolescents reported waiting until severe symptoms appeared before seeking care. While some participants reported being turned away from clinics which failed to identify/diagnose their TB, other participants reported being diagnosed with viral or bacterial infections including bronchitis and pneumonia and being put on treatment for these before receiving a TB diagnosis. This delayed TB treatment initiation by up to 2 months.

**Table 2:** Quotes of participants prediagnostic and treatment pathway experiences

Prediagnosis and treatment pathways	Participant	Quote
Delaying healthcare seeking	Late adolescent male participant, DS-TB	“Firstly, I stopped eating and then started losing weight and started sweating more and more and my chest—I started coughing a lot. Then in between, I had chest pains, and I just didn’t know what was actually happening in my body (…). One evening I remember, I just couldn’t sleep and then eventually I fell asleep and the next morning I woke up and my sweater was soaking wet, you could literally dry it out. I took it to my grandmother, and she got a shock, and then she told me, ‘OK, you know what? Let’s go to a private doctor then.’ She gave me money and then I went to a private doctor (…). He told me he was going to do a couple of tests, but from what I’m telling him, he has a few things in mind, but the main thing that stands out is TB. But he told me, ‘I am going to have to go to a TB clinic to go get tested.’ (…) Then he did that test and then he said, ‘Yes, that just confirmed that you have TB.’ (…) I was heartbroken about TB. How could this happen to me?”
	Early adolescent male participant, DS-TB, HIV-positive	“I'm not a person who likes to be indoors. Most of the time, I like to go out with my friends. So, I suddenly appeared to like being at home. I was always tired and sleeping. Then they noticed that I was coughing up blood, and now it is thought to be TB. I keep coughing up blood. And it became serious, then I gave up and went to the clinic. Maybe if it hadn't gotten serious, I wouldn't have gone to the clinic. (…) I started feeling sick in mid-September 2020. (…) I only went to the clinic on the 18th of November 2020. (…) When I came back on the 20th, the nurse told me that I was diagnosed with TB, and I started taking treatment.”
	Early adolescent female participant, MDR-TB	"I was sweating at night, I was coughing. I had headaches. I went to the [local] clinic. They gave me allergy medicine, and I lost weight. I cried to myself, not wanting to go to the clinic because of the injection. I was told to cough, and I coughed. I was told to come back and get the results. We went there, I was told I have TB.”
	Early adolescent female participant, MDR-TB	“It was the 14^th^ or 15^th^ of December; I was starting to cough a lot. After 2 weeks, when I was coughing, my chest was painful, and I had pain under my armpit. My mother said it is because I am always in the streets. My mother told me to show my hands. She said, ‘No, I don't have TB’, but she was suspecting that I have it. I think 2 months passed, and my mother said that I am sick. I should go to [the local] clinic. Then on the 28th of March, I went to the [the local] clinic, and they said I must make an appointment. My stepfather told my mother she must take me to the clinic because I was coughing up black mucus. My mother used to ask me if I was not sweating at night, and I said no because I did not want to go to the clinic. Then my mother took me with her to [another] clinic. They ran some blood tests, and I was also tested for Covid. I was told to come back the following week on Tuesday. When I returned on that Tuesday, I was told I have resistant TB. (…) On the following Tuesday, they told me to go for X-ray. The doctor told me that on the side that is painful when I am coughing, it is because my left lung has a hole, and the right side of my lungs are ruined and only half is healthy. They told me I will have to start treatment on the same day, the 15th of April. Then the doctor explained to me what resistant TB and the doctor has told me that I was infected by someone else because my mother thought TB was caused by smoking and drinking.”
Misdiagnosis/missed diagnosis	Early adolescent female participant, MDR-TB	“I was sweating at night, lost weight and I didn't want to eat. Then at home, they gave me medicine bought from a pharmacist. I was first given medicine before going to the clinic. The medicines did not help, so I went to the clinic. It was my mother and my aunt who advised me to go to the clinic. At the clinic, I was always turned away, and they said they couldn't see anything. After being turned away several times, we didn’t want to come back and I was feeling okay. Then the clinic called me this year; they told me I have TB (…). They came to my home by car to tell me that I have TB and I started treatment.”
	Late adolescent participant, MDR-TB, HIV-positive	“I started to get sick for the second time in 2021. We were drinking a lot with my friends (…). I woke up with pain; I thought it was a sore throat. My body was tired, I still didn't think it was TB. So, I kept having bad dreams, and at church, the Pastor said that something was following me, like evil spirits. Then I went to another father. He washed me and gave me medicine, and I was fine. The father is a traditional healer. (…) I felt that something was not right; I was shivering. (…) Then in the second week, I wasn't interested in my friends, and I didn't want to admit that I was sick. I forced myself, even when it came to smoking. I didn't smoke as much as I used to. So, my father noticed that (…) I always have white eyes and he saw that I was always tired. He said I should go to the clinic. (…) I said I'm alright, I won't go to the clinic. There is another place, you know, that they ask you to take an X-ray, but they can't test one for TB without the HIV test first. So, my father didn't want me to test because they were local people. So, we went to a small mobile clinic, and they told us the results of the X-ray—that I was right. (…) I continued to drink and smoke. I felt the next day that I'm not alright, I'm coughing. My father told me that he will no longer allow me to make my own decisions and he will take me to the clinic.”
	Late adolescent female participant, MDR-TB, HIV-positive	"I felt like I had a chronic flu. I was coughing. Then, after 2 weeks, my mother took me to the clinic because I had night sweats. When I came to the clinic, nothing was found. I was taken for an X-ray [at another] clinic that same July. After the X-ray results came out, they said I have TB. Then I started treatment.”
	Early adolescent male participant, DS-TB	“The first time I went to the doctor (…) not to the clinic (…) my sister and I were in the [name of private] hospital (…) because I was sick. The doctor said I had pneumonia, but it wasn’t like that. So [later], they said it was TB.”
Diagnostic pathway	Early adolescent female participant, DS-TB	“Like when I found out I have TB (…), that was 2 months ago, January month that I began to cough. I began to sweat at night, I felt very fatigued, I didn’t want to eat (…) but I didn’t go [to the clinic] in January (…) I only went in February. (…) We went to our clinic (…) I spat [produced sputum] and then I came back after 3 days so they could give me my results. (…) They didn’t send me anywhere, they just gave me pills and I went home. (…) When I [first] felt [TB symptoms] my mother gave me (…) capsules. I don’t know what capsules those were, but it helped with the sweating.”
	Late adolescent female participant, MDR-TB, HIV-positive	“The last time I was sick I went to Green World pharmacy. The consultant put me on a scan, and then they told me that my lungs are not okay, and I have a cold. They gave me herbs to help me, but because I could not afford the herbs anymore, my cousin advised me to go to the clinic because I will get free medication. When I went to the clinic to get tested, I waited for my results for 3 weeks. Then the doctor called me and told me the results, and I started treatment immediately. Sometimes I go to [a traditional health practitioner] to do ‘intlahlubo”*.

Late adolescence (18-21 years); early adolescence (12 - 17 years).

* Intlahlubo is a diagnostic process used by traditional health practitioners, to divine illnesses and their root causes by channelling ancestral communication.^50^

DS, drug-susceptible; MDR, multidrug-resistant; TB, tuberculosis.

Although all the adolescents recognised traditional, alternative and spiritual healers as playing a role in their communities, only a few consulted these healers for their TB symptoms. One participant, whose mother was a practising traditional healer, had taken traditional medicines for a prior ailment, but did not take traditional medicines to treat their TB. Another participant reported consulting a traditional healer to exorcise evil spirits that his pastor divined were the underlying cause of his illness. The participant reported going to a specialised herbal pharmacy for some time before resorting to a local public health facility. A few adolescents reported seeking care at privately run pharmacies as their initial care providers—primarily in pursuit of symptomatic relief. When participants’ symptoms persisted, some sought care from private doctors, but most sought care from local PHC facilities. Although some PHCs could diagnose TB, some adolescents were referred to secondary hospitals for further diagnostic testing, including X-rays. Once adolescents had received a TB diagnosis, most received ambulatory care, though some were hospitalised for short stints at either a specialised TB hospital or at a tertiary hospital in the City of Cape Town municipal area.

### Experiences of diagnosis and treatment initiation

Participants shared similar experiences when receiving their diagnoses, regardless of age, resistance pattern of *Mtb* strain or gender (table 3). While participants reported being tested for TB at their local PHC, by producing sputum and blood samples, some participants reported having to wait for confirmation through a chest X-ray—often conducted at a different health facility. Adolescents with MDR-TB sometimes initiated TB treatment for a few days before their drug resistance profile was confirmed. This was often due to adolescents’ local PHC facilities not having the necessary equipment, such as X-ray machines or GeneXpert instruments, to diagnose TB or MDR-TB, and they were referred to another clinic or hospital for these tests.^36^

**Table 3:** Quotes of participants’ diagnostic and treatment initiation experiences

Diagnosis and treatment initiation	Participant	Quote
Knowledge of TB and diagnostic process	Late adolescent female participant, DS-TB	“The symptoms started to show a week after [my cousin] passed away [from TB]. (…) I walked to school, it was normal, but while I was sitting in the exam room, done writing, I tried to breathe and I took a deep breath, and I felt this pain down my back, where my lungs are, and I stretched. And then I knew, something isn’t right here. Because every time I have TB, I get this symptom, pain behind my back. I decided to go home. (…) I knew ‘no, this is now TB that’s back again’. Because of this pain in my back, I couldn’t even laugh. (…) I went to the clinic, and I decided they should test me. (…) So, then I came back, and I went for my results, and they said I should have a seat, and then I knew, ‘nah, it’s positive’.”
	Late adolescent male participant, DS-TB	“[My family] said it was something they always knew, because I am not the first one at home to have TB because my older sister died of TB (…). They know that the TB treatment takes 6 months but if you don't take your treatment appropriately, then it progresses to MDR-TB. So, they were not shocked when I told them. (…) I didn't feel embarrassed because they always said at home that they suspected TB, not HIV. I wasn't shocked or traumatised.”
	Early adolescent female participant, DS-TB	“TB is not a deadly sickness; there is a cure, but I don’t actually know what it is. When I found out I have TB. I don’t know, I wanted to cry. I expected it. I had the symptoms and all that, but to hear it.”
	Late adolescent male participant, DS-TB	“My mother was a TB and HIV counsellor, so she basically told me everything about these things. (…) Well, I would say [other people] in the household [with TB] it would be my sister, the eldest one(…), my aunty, (…) my grandma and the other [aunt] (…) they had it. (…) My aunty, she had TB last year or the year before that.(…)Then my brother got [TB] and he finished his treatment. (…) As soon as he finished [his treatment] Then I had [TB] again. (…) The nurse said that there is a possibility of a carrier in the house. So, I mostly think it is my uncle because he smokes everywhere and he would say stuff like, (…) he is immune, he can’t get TB (…). It was in March that I was like just coughing for a week. I was sweating at night. (…) Then I just told my mother I don’t want to go to school now, it’s already a risk with COVID. So, I said to her that I’m going to go for a test. And then I went to go spit and I started my treatment.”
	Early adolescent female participant, MDR-TB	"In the beginning, I was diagnosed with TB. I wasn't very sad because I knew that I would be fine after taking my pills because my cousin finished his pills, and he was alive.”
Psychosocial experience of diagnosis	Late adolescent male participant, DS-TB	“After seeing a doctor and being referred to the TB clinic, I was heartbroken.(…) I knew the symptoms and things, but I wasn’t fully educated, then I found out the treatment was 6 months! I thought I don’t know how I’m going to get through these 6 months. (…) I was very scared, to be honest.”
	Early adolescent male participant, DS-TB	“I went back with [my] mom to the clinic because she was also not expecting the results to come back positive for TB. She then started taking it seriously after the results as she was quite shocked.”
	Early adolescent female participant, DS-TB	“My mom wasn’t with me when I got my results. I was alone. For me, it was, what are my friends going to say? Are they still going to be friends with me? Will they abandon me? (…) So, they knew I went for the test, and they asked me, and I told them, ‘Yes, I have TB.’ (…) They were always normal. I said they need to go and test, and they said no, they are not going to test themselves.”
	Early adolescent female participant, MDR-TB	“I felt very bad. I could not believe that I have TB. When my mother found out that I have TB, she was very upset because she thought that I am a smoker, but the doctors at the clinic told her that I am not smoking.”
	Early adolescent male participant, DS-TB	“I was happy because it was like a solution to all [the symptoms] I’ve been experiencing.”
Treatment initiation	Early adolescent male participant, DS-TB	“It was before the weekend, on a Friday. I was held back at school, and I would have gotten to the clinic too late to get my treatment.”
	Late adolescent female participant, DS-TB	“It wasn’t difficult to take [my treatment] but was difficult to go [to the clinic]. When I [was diagnosed with] TB, it’s because I was lame. I couldn’t walk by myself; I fell over. (…) A person sometimes gets tired of your treatment. (…) It’s too big, it doesn’t go down well and it’s difficult. Especially when you become ill, and you are really seriously ill. Then it is very difficult to walk to the clinic and (…) you just don’t have food in your body. You throw the food up (…). So, you don’t have energy to go to the clinic.”
	Early adolescent female participant, DS-TB	“I just forgot. I went out, and when I came in, my mom asked if I took my pills. I said ‘no,’ and then I took them. I didn’t have the motivation to go out, to eat, to see anything, just to lay and watch TV.”
	Late adolescent male participant, DS-TB	“Because this stuff makes you tired. (…) I walk to the clinic so your lungs aren’t the same and when I go there, I take my tablets and then it’s like hard, it’s like hard, the walk back because the stuff is like, it’s tiring, there’s times where my muscles pull stiff, so those kind of things. (…) Well obviously that stuff is large, the tablets, and there were times where I actually choked on it.”
	Early adolescent female participant, MDR-TB	"I felt bad when I was told that I have TB because we take many pills every day. (…) I never liked this, it never felt good because I was going to take pills and I didn’t know how to take a pill (…) I blamed my sister, I thought it was her.”
	Early adolescent female participant, MDR-TB	“They told me to stop going to school for 3 months. (…) The doctor advised my mother to not live in [our community] anymore because it’s dirty. Then we moved to rent a flat in (a different community) for 3 months and we came back when I was feeling better. My stepfather is working for a law firm. He then went to speak to the [school] principal and told him/her that he does not expect any ill treatment towards me, and that made me not experience any stigma from school. I take 18 tablets on Monday, Wednesday and Friday. Then Tuesday, Thursday, Saturday and Sunday I take 16 tablets.”

Late adolescence (18-21 years); early adolescence (12 - 17 years).

DS, drug-susceptible; MDR, multidrug-resistant; TB, tuberculosis.

Adolescents—even those who suspected they might have TB—were either shocked or saddened by their diagnosis. Adolescents reported an MDR-TB diagnosis as more distressing than a TB diagnosis. This may be related to the language used by health workers or language pervasive in their communities suggesting that MDR-TB is more dangerous than DS-TB. Having a prior TB episode or household/family members who had had TB appeared to mitigate some of the distress associated with a TB diagnosis. Participants reported that since they had seen others healed of TB, they would also become well if they adhered to their treatment. Additionally, having had a prior TB episode or household/family members who had had TB seemed to hasten participants’ healthcare seeking behaviour. One participant, a 16-year-old male participant with DS-TB, mentioned being relieved at his diagnosis, explaining that he now had an explanation for the symptoms he was experiencing.

A few participants reported attending the health facility alone for their symptoms and were alone when receiving their diagnosis. Adolescents reported feeling overwhelmed by their diagnosis and unsupported by family members. In some cases, adolescents’ families only took their symptoms seriously when adolescents returned home with their TB diagnosis and treatment in hand. Adolescents were sometimes blamed for their TB diagnoses—with family members accusing them of participating in what they perceived to be risky behaviours.

For many of the participants, the emotional and social implications of having TB were substantial. This was true regardless of their *Mtb* resistance profile. Participants anticipated being stigmatised because of their TB diagnosis, which often led participants to isolate themselves, impeding social support and sometimes delaying treatment initiation. As a result, some adolescents sought care from clinics outside their local community PHC facility. Once initiated on treatment, these initial fears, anticipations and related choices led to downstream challenges to ongoing treatment and care. Thus, TB can cause significant community stigma, negatively affecting adolescents’ interactions with healthcare systems throughout their TB treatment.

### Adherence to care and interactions with the health system

Adolescents reported that immediately after treatment initiation, they were expected to report to the clinic every day for the first 2 weeks where they were observed taking their treatment (table 4). Subsequently, adolescents diagnosed with MDR-TB had to visit their local health facilities on a weekly basis for a period of up to 3 months, prior to receiving a month’s supply of treatment. The frequency of clinic visits to receive treatment appeared to participants to be applied inconsistently, with the criteria used to make decisions about when to apply direct-observation therapy unclear. This led to a sense of injustice and mistrust by participants who implied that they were being ‘singled out’ as undeserving of autonomy in their care. Other participants reported being required to go to the clinic every day for over a month. Moreover, those who self-disclosed as being poorly adherent were compelled to revert to directly observed therapy, following a period of collecting their treatment only once a month—again, disincentivising honesty.

**Table 4:** Quotes of participants’ treatment adherence and health system experiences

Adherence to care and interactions with the health system	Participant	Quote
Treatment adherence experiences	Late adolescent female participant, DS-TB	“You know, I was irritated, because why can I have TB again. (…) my mother was rude and whatever, but I wasn’t worried about that.(…) there was a few [days that I missed treatment] because sometimes when I take my treatment, when I took it in the morning, then I’m forgetful. But you know I have son that keeps me busy, so by the time I realise that I didn’t take my medicine, it’s late. (…) I take it that time (when I remember). (…) But I was very glad, so I took the tablets until 2nd November, then I took a sputum, the next month, the 28th of December, and then it actually came back negative. (…) My thing is nobody actually helps me because, if you’re sick, or if you’re ill, you as a person wants to get better. So, you should know you should take your tablets. You don’t need someone to tell you, ‘Hello, remember to take your pills.’ No, it’s your responsibility. So, nobody did that for me.”
	Late adolescent male participant, DS-TB	“From there I went to the TB clinic (…). I got there and I explained to them. I went for a sputum and 2 days later I came back (for my results]. (…) They explained to me that I currently have TB, so they are going to have to start me on treatment for the first week. During the first 2 weeks I shouldn’t be in contact with anybody. So, I have to come every morning to take my medicine so that they can monitor me taking the medicine as well as any side effects that the medicine might have. (…) I have this thing where I’m tired most of the time from the tablets. (…) The first point that I want to bring up is that I stopped my treatment. (…) It was the best feeling ever, the day that I went to go and fetch my last meds. And I thought that I must get two or more weeks [of medication], then (the nurse just told me, ‘Here’s your last packet, you don’t have to come in again. Enjoy, look after yourself.’ I just lit up; I was so happy I got through the 6 months. (…) You get used to going to taking medication on a daily (basis), going to doctors, so you build that type of responsibility.”
	Late adolescent female participant, DS-TB	“I could do nothing (…) I took a few spoons of food at night then vomited. I laid alone in bed and coughed, and it would just be slime that came out. I had no connection to another person.”
	Early adolescent female participant, MDR-TB	“I missed an appointment once to fetch tablets because my mother was at work, and I went to the clinic the following day with my mother’s friend. I missed treatment for that 1 day. My mother’s friend is supportive; she steps in when my mother is not available. We take treatment in the morning; we did not experience any side effects. My mom motivated us to take treatment and the importance of doing so.”
	Early adolescent female participant, MDR-TB	“I started treatment on the 5th of March. I missed taking treatment several times. I would take it, but sometimes take half of the treatment and throw the other half away. The tablets were annoying because it was difficult to ingest them, and I throwed them away, and no one knew what I was doing. My mother found the tablets in the bin, and she got worried, and she asked for me to be admitted [to a TB hospital] so that I can take my tablets properly. (…) When I was at the hospital, I started taking the treatment again, and I was afraid of dying. I committed myself in taking my tablets because I was afraid of dying. I started treatment again in August, and I am taking 15 tablets. (…) My mother was heartbroken when she found the tablets in the bin, and she is still supportive and also my father.”
	Early adolescent male participant, MDR-TB	When asked about who supports his treatment adherence, the participant responded:Participant: “I go alone to the clinic alone.”Researcher: “Do you go alone to hospital and pharmacy as well?Participant: “Yes.”
Health systems interactions	Late adolescent female participant, DS-TB	“The first time I was here, (one of the study doctor’s) was like, ‘you’ve had TB three times,’ and she checked my lungs, and she was so shocked because if you’ve had, or if someone’s had TB thrice, there’s obviously going to be a problem with your lungs. But every time I breathe in and she’s listening to my lungs, she told me, ‘There’s actually nothing, your lungs don’t sound like a person that has experienced TB thrice.’ She’s not getting anything because I’ve had x-rays on my lungs, everything came back cleared. (…) My thing is that maybe I have a lung problem or whatever, but then every time I do the sputum, it comes back with TB. But I’m telling you now (…) you know what I said, my symptoms last year were, the pain in my back and then it was just gone automatically. (…) Sometimes I feel they take the sputum, they put in my ID number, and it pops up that I was someone that had TB before. So, they have the results and the records and everything. So, I feel like maybe they just wrote there that I’m positive. Yes, because, you know, it’s like they saw me like, first, second, third, oh this is the third time. ‘Oh, she’s had TB before.’ And then after a month, it comes back negative, so my thing is, I really want to know what this is coming back to. And trust me, [the doctor] didn’t tell me anything.”
	Late adolescent male participant, DS-TB	“They kept me there and I went for therapy. They call it therapy, where this woman explained to me what TB is and how it can be treated, and things to do and not to do. At that time, it really helped.”
	Late adolescent male participant, DS-TB	“I am satisfied with the information about TB that I received from nurses, doctors and counsellors.”
	Early adolescent female participant, MDR-TB	“People from [my local clinic] came to my home to tell me that they had found I had TB. (…) When I went to the doctor, the doctor said to me, it’s people like you who die from TB because you do not want to take your treatment.”
	Late adolescent male participant, MDR-TB	“I ran out of tablets on the 29th of July, and I was not taking tablets for about 2 weeks now and until the 10th of August, and this is because I ran out of some tablets, and I was afraid to take short number of pills. I thought I can only go back to the clinic on the specific date I was given, and I’m afraid to go to the clinic when it is not my date’’.
	Late adolescent male participant, MDR-TB, HIV-positive	“I prefer going to a private doctor, because there are few people there, my stepfather accompanies me and pays, at the local clinic there is always a queue and it’s embarrassing.”

Late adolescence (18–21 years); early adolescence (12–17 years).

DS, drug-susceptible; MDR, multidrug-resistant; TB, tuberculosis.

Adolescents’ ability to adhere to their treatment was complicated by their inability to collect their medication from the clinic—mostly due to practical logistical issues. Some adolescents cited being so unwell that they were physically unable to walk to the clinic to fetch their treatment. A lack of transport or costs related to transport, parents being at work or being at school until after the clinic closed were other reasons adolescents provided for not being able to collect their treatment themselves. This was true for adolescents, regardless of their resistance profile.

The treatment itself was also frequently reported as an obstacle to adherence. For example, adolescents cited the high pill burden of treatment and adverse effects of the treatment. Some adolescents described the adverse effects of the treatment as so severe that they did not go to school for 2 weeks. The pill burden and adverse effects of treatment were particularly challenging for adolescents with MDR-TB and those co-infected with TB and HIV, for whom the treatment is more complex. Forgetting to take the medication or adherence being deprioritised due to improved health or the prioritisation of other household/personal goals also contributed to poor/inconsistent treatment adherence among participants. Some adolescents reported receiving adherence support from caregivers and friends, while others reported that adherence was for their own good—they were self-motivated to adhere to treatment. Older adolescents (aged 16–19 years old) appeared to receive less social support and were more isolated or decided to isolate themselves from others than younger adolescents (aged 13–15 years old). It could be that older adolescents are seen as responsible for their own well-being, whereas younger adolescents continue to be cared for by their guardians.

Most participants described their interactions with healthcare workers as limited, interacting principally at diagnosis and treatment initiation. There was some additional interaction when adolescents collected their medication from PHC facilities. Despite frustration at the health systems’ shortfalls at diagnosing and monitoring their TB, adolescents reported positive interactions with health workers, saying that they felt cared for. Some participants reported being provided with sufficient education on TB and its treatment to feel empowered to care for themselves, while others reported not receiving an explanation for their disease, treatment and symptoms. However, adolescents with poor adherence to their treatment reported that health workers spoke to them in a confrontational and pejorative manner. A frequent source of negative sentiments among adolescents towards health services was in how their adherence was monitored, impinging on their sense of autonomy. A few adolescents bemoaned presenting at local facilities for fear of being seen by others and the long queues.

## Discussion

We found that adolescents had varying experiences of healthcare facilities and health workers, with some participants receiving empathic care and swift diagnoses, while other participants experienced delayed diagnoses and inadequate care. This variability did not seem related to the adolescents’ age, gender or their *Mtb* resistance profile. Our findings indicate that adolescents diagnosed with DS-TB and MDR-TB experienced a range of social and psychological issues along their treatment journeys, particularly in relation to treatment processes, health system access and healthcare and social support. However, participants with MDR-TB tended to experience more complicated treatment journeys—interrupting treatment more frequently, suffering worse adverse effects, and potentially experiencing more stigma. We have previously reported that adolescents in this cohort lacked social support and faced high levels of stigma and depression, negatively impacting their healthcare access—the focus of this study.^37 38^

Participants in our study reported challenges receiving a diagnosis—not only being misdiagnosed and given the incorrect treatment, but also being referred to and from multiple health facilities before being correctly diagnosed with MDR-TB or DS-TB. Studies have demonstrated that in adolescent accounts of their health needs and symptoms, they feel marginalised and they experience health workers as non-responsive.^20^ As seen in ours and other studies, fear of discrimination and pejorative language used by health workers or others in health facilities could deter patients from seeking and/or adhering to treatment.^39^ Other studies have also found that adolescents’ reticence to attend care after diagnosis also poses a threat to treatment adherence and health outcomes, especially among adolescents with MDR-TB.^2 29 40^

As in other studies, adolescents in our study bemoaned the inconsistent application of direct observation therapy and related adherence ‘support’, which required facility visits, causing interruptions to everyday routine.^2^ Interruptions to schooling and work commitments accompanying long waiting periods at clinics have also been reported in other research.^20 30^ While some research has suggested that adolescents might prefer to receive care at a community or household level,^41^ a recent study on adolescents’ preferences for TB preventive treatment suggests that adolescents preferred to receive this treatment at their local clinic to avoid potential stigma and wanted fewer clinic visits with a shorter treatment duration.^42^ Together, these data suggest that adolescents should be involved in decision-making regarding their treatment regimen and healthcare processes.

Unlike a study among adolescents living with HIV, which reported that families provided them with instrumental support (comprising transport to PHCs and reminders about pill-taking), our participants reported often having to travel to the clinic alone, frequently by foot.^43^ Participants in our study indicated that a lack of resources, with the prioritisation of work opportunities over accompanying/transporting them to PHCs, may have underscored this lack of support from family members. Another study conducted in South Africa among people living with HIV revealed that disease management occurs among a complex of other household responsibilities which may disrupt treatment access and adherence.^44^ Adolescents in our study also complained about not being emotionally supported when receiving their diagnosis, as well as during treatment adherence. Other studies found that providing psychosocial and material support to people affected by TB greatly improved treatment success.^45 46^ TB treatment remained a major barrier to adherence among our participants. Pill burden and adverse effects of drugs were oft-mentioned challenges to adherence, especially among participants with MDR-TB and TB-HIV co-infection. A groundswell of research showcasing the challenges involved in adhering to TB treatment has spurred the development of shorter regimens, with a lower pill burden and fewer adverse effects for children and adolescents.^47 48^

Our study was conducted at a single site, limiting transferability. Although adolescents in this study received their treatment and care through the national TB programme, they also received additional care by participating in this study, potentially obscuring the extent of adolescents’ care needs. Data collection also occurred in 2021 and 2022, during the COVID-19 pandemic which had a dramatic impact on health services, including TB treatment and care services. Strengths of our study include involving a wide age range of adolescents, from different ethnic backgrounds and with different forms of TB disease with and without HIV co-infection. We also had multiple interactions with each participant over a long period, with iterative refinement of data collection and processing built into the analytic process to ensure rigour.

The need to offer health services tailored for adolescents has long been recognised.^49^ However, as our research suggests, it is important that we work with adolescents to understand which models of care they prefer, which may include home-based or community-based services rather than facility-based services. Our research also indicates that further research needs to be done to better support adolescents’ (and their family members’) knowledge of TB disease and management towards improving their social and treatment adherence support. Additionally, more work needs to be done to alert health workers to adolescents’ specific TB care needs. More research is needed to improve diagnostic tools to ensure timely diagnosis and treatment initiation for adolescents. Despite recent advances in TB treatment, which includes shorter, easier to administer drugs with fewer adverse effects, it is important to note that many of these drugs have not yet been approved for use in children and adolescents, and even where they have been approved, they are not universally accessible.

## Data Availability

Data are available upon reasonable request.
