# Supplementary material for: Understanding the relationship between adolescents with tuberculosis and health services: an indepth qualitative study from Cape Town
Source: BMJ Open. 2025 May 24;15(5):e094295. doi: 10.1136/bmjopen-2024-094295 (PMC12104947; doi:10.1136/bmjopen-2024-094295)
Supplement: Supplementary file 1 [file bmjopen-2024-094295.reviewer_comments.pdf]

PEER REVIEW HISTORY

BMJ Open publishes all reviews undertaken for accepted manuscripts. Reviewers are asked to complete a checklist review form and are provided with free text boxes to elaborate on their assessment. These free text comments are reproduced below.

ARTICLE DETAILS

Title (Provisional)

Understanding the relationship between adolescents with tuberculosis and health services: an in-depth qualitative study from Cape Town

Authors

Wademan, Dillon; Hoddinott, Graeme; Kavalieratos, Zara; Mlomzale, Mfundo; Marthinus, Arlene J; Jola, Lucia N; Jacobs, Stephanie; Mcimeli, Khanyisa; Seddon, James

---

VERSION 1 - REVIEW

**Reviewer**	**1**
**Name**	**Rosnani, Zakaria**
**Affiliation**	**University Sains Malaysia - Health Campus, Department of Family Medicine**
**Date**	**19-Oct-2024**
**COI**	**None**

Well written with the objectives and result clearly presented.

Method- HIV testing done after TB diagnosis or simultaneously during TB investigations?

Explain the inclusion criteria- within 14 days of receiving treatment.

Any extra interviews for adolescent with HIV co-infection and MDR TB compared to DS TB.

Discussion- to elaborate further in the adolescent with HIV- co infection- is there any counselling done beforehand with regards to the implication on insurance, prognosis and treatment duration.

Emotional impact- as being call dirty for having TB and has to move out-need to relate to the community stigma and belief system.

Are they being neglected by their friends and family

**Reviewer**	**2**
**Name**	**Lisum, Kristina**

Affiliation STIK Sint Carolus

Date 23-Oct-2024

COI None

---

1. consistency of terminology that used, such as teen/adolescent

2. the statistical metod that used for this qualitative research?

3. the result : what is the theme or sub theme of this study?

4. date of ethics approval

5. the objective not clearly defined.

Reviewer 3

Name Rodrigues, Rashmi

Affiliation St Johns Medical College, Community Health

Date 16-Dec-2024

COI None

First of all, I congratulate the authors on the work that they have described in this manuscript. It is very interesting that to understand TB and health systems from the adolescents perspective.

As requested here are my comments on the methodology that may be used to provide a clearer understanding of the work done:

1. Under the study setting it would be good to know where the diagnosis of DR TB takes place (i.e., at which level in the healthcare system). How is this different from DS TB diagnosis?

This is especially so as the objective is to understand the interactions between the adolescents and the healthcare system.

2. Please describe the types of treatment regimens that are available and prescribed to adolescents along with the duration of treatment.

3. What are the systems in place for identification of ADRs, pill refills and adherence support. Is there any counseling provided?

4. What is the system for inpatient care for DR TB?

Teen TB: It is easy to mistake this for the study design- hence please state upfront that the current study (manuscript) is part of a larger study titled, 'Understanding the biology...) this is not very clear at the moment.

Study design and sampling: This is done well. However please clarify why only 20 participants were selected for participation. Also, the paragraph indicates 19 participants but the table has 20- while this has been described in the abstract the same clarity is necessary in the methods.

Data collection process:

1. How was data recorded? Given the varied methods used during data collection- it was probably necessary to audio record interviews and use photographs of body mapping activities- kindly indicate these. Was consent taken for recording these procedures?

2. Please indicate the time taken for the interviews.

3. Where were the interviews conducted? was someone else present at the interviews or were the adolescents alone with the interviewer? Was there privacy?

Data analysis: While it is understood that a deductive analysis was done, please describe the analytical approach used (thematic/content/framework).

Ethical considerations: Please indicate if consent for this study was obtained under the main study or if reconsenting was done.

Additional aspects: as the analysis is deductive kindly indicate the theoretical framework/ basis for the study.

Limitations (from a methods perspective):

1. The sample size limitation does not apply to this study as it is qualitative.

2. It would be good to also describe the limitations/ methodological issues in terms fo trustworthiness, i.e., transferability, credibility, dependability, and confirmability.

3. An additional note on reflexivity will be helpful.

VERSION 1 - AUTHOR RESPONSE

Reviewer 1's Comments:
In the table below, we respond to the Dr. Zakaria Rosnani's comments (left column) and indicate the specific changes we made to the manuscript (right column).

Comment	Response
1. Well written with the objectives and result clearly presented.	Thank you.
2. Explain the inclusion criteria- within 14 days of receiving treatment.	We have provided further clarity to the inclusion criteria (see lines 136-140):<br><br>"Adolescents were included in the TB arm of Teen TB if they were adolescents (10-19-years-old) with newly diagnosed pulmonary TB bacteriologically confirmed on sputum (Xpert MTB/RIF positive or culture-positive), with or

	without HIV co-infection, regardless of drug resistance testing profile, and who were within the first 14 days of treatment, a cut off that ensured we only recruited adolescents soon after TB diagnosis and treatment initiation.”
3. Any extra interviews for adolescent with HIV co-infection and MDR TB compared to DS TB.	Thank you for the opportunity to clarify. No, we did not purposefully conduct more interviews with adolescents living with HIV co-infection or MDR-TB.
4. Discussion- to elaborate further in the adolescent with HIV-co infection- is there any counselling done beforehand with regards to the implication on insurance, prognosis and treatment duration.	We appreciate the opportunity to provide further clarification. Any adolescents with a new HIV infection diagnosed during this study were provided with counseling and referred to routine services for evaluation and initiation of ART. Adolescents with an existing HIV diagnosis would have received counseling before initiating ART at their local healthcare facility. We have added a sentence to explain this in the methods (see lines 141-142):<br><br>“HIV testing was performed if the individual did not have a negative HIV test in the preceding 6 months, by a trained HIV counsellor, following informed consent, and with pre- and post-test counselling.”
5. Emotional impact- as being call dirty for having TB and has to move out-need to relate to the community stigma and belief system.	Thank you. We have added the following sentence to the Findings section (see lines 252-254):<br><br>“Thus, TB can cause significant community stigma, negatively affecting adolescents' interactions with healthcare systems throughout their TB treatment.”<br><br>To clarify, this manuscript focuses specifically on adolescents' experiences with healthcare systems. A companion manuscript, also using this dataset, explores the often highly negative psychosocial impact of TB on adolescents. For interest’s sake, we include a link to the manuscript: https://doi.org/10.1371/journal.pgph.0003539
6. Are they being neglected by their friends and family?	Thank you for raising this important question. As we have highlighted above, the companion manuscript explores adolescents’ psychosocial experiences of TB in more detail: https://doi.org/10.1371/journal.pgph.0003539

Reviewer 2's Comments:
In the table below, we respond to the Dr. Kristina Lisum's comments (left column) and indicate the specific changes we made to the manuscript (right column).

Comment	Response
1. Consistency of terminology that used, such as teen/adolescent	We have removed all instances of the word 'teen' and replaced it with adolescents, apart from specific reference to the Teen TB study, which cannot be altered as it refers to the study per protocol.
2. The statistical method that used for this qualitative research?	Statistical methods were not used. This is an entirely qualitative study. We assume the reviewer meant analytic process. Please see lines 175-185 where we describe the analytic process.
3. The result : what is the theme or sub theme of this study?	Apologies. We do not understand this question. Overall, the study sought to describe adolescents' interactions with the health system. The manuscript details this through three thematic topics that resemble the TB care cascade. Specifically, it describes: (1) adolescents' pre-diagnosis and treatment pathways, (2) adolescents experiences of diagnosis and treatment initiation, and (3) how adolescents adhere to care and their experiences interactions within the health system.
4. Date of ethics approval	Apologies for the oversight. We have included the date of ethics approval. See line 189:<br><br>First approved on the 25 February 2020
5. The objective not clearly defined.	Thank you for the opportunity to clarify. We have edited the end of the background section as follows to more clearly define our objectives. See lines 108-111:<br><br>"Specifically, our objectives were to (1) describe adolescents' experiences of their TB diagnosis and treatment pathways, and (2) to identify health service delivery issues that are linked to both positive and negative experiences of adolescents along their care journeys."

Reviewer 3's Comments:
In the table below, we respond to the Dr. Rashmi Rodrigues's comments (left column) and indicate the specific changes we made to the manuscript (right column).

Comment	Response
1. First of all, I congratulate the authors on the work that they have described in this manuscript. It is very interesting that to understand TB and health systems from the adolescents perspective.	Thank you.
2. Under the study setting it would be good to know where the diagnosis of DR TB takes place (i.e., at which level in the healthcare system). How is this different from DS TB diagnosis?<br>This is especially so as the objective is to understand the interactions between the adolescents and the healthcare system.	Thank you for this insightful comment. We have added the following detail to the setting, explaining the general DR-TB diagnosis process (lines 121-123):<br><br>"Over the study period, diagnosis at primary care was primarily through Xpert MTB/RIF on expectorated sputum samples, with treatment initiation and ongoing care at that clinic for both DS- and DR-TB."
3. Please describe the types of treatment regimens that are available and prescribed to adolescents along with the duration of treatment.	Thank you. We have provided the following information regarding adolescents' treatment regimens in lines 123-127:<br><br>"At the time of the study, the standard of care treatment for adolescents with DS-TB in the Western Cape, comprised a 2-month intensive phase with isoniazid, rifampicin, pyrazinamide, and ethambutol (2HRZE), followed by a 4-month continuation phase with isoniazid and rifampicin (4HR). Adolescents with MDR-TB received injectable-free treatment, with durations ranging from 9 to 20 months, depending on their drug resistance profile."
4. What are the systems in place for identification of ADRs, pill refills and adherence support. Is there any counseling provided?	Thank you. This is a similar question to Reviewer 1's fourth question. Adolescents do not receive routine counselling after receiving their diagnosis and initiating treatment, nor is there routine adherence support.<br><br>We have however, added the following sentence to clarify counselling provided to adolescents (see lines 140-142):<br>"HIV testing was performed if the individual did not have a negative HIV test in the preceding 6 months, by a trained HIV

		counsellor, following informed consent, and with pre- and post-test counselling.”
5.	What is the system for inpatient care for DR TB?	While we did not explore this in detail, in the Western Cape, where this study was conducted, most patients are managed as outpatients. If they need inpatient care for complex MDR-TB, additional medical needs or complex social needs they are admitted to specialist TB hospitals until stabilized at which point they can be transferred back to primary care.
6.	Teen TB: It is easy to mistake this for the study design- hence please state upfront that the current study (manuscript) is part of a larger study titled, 'Understanding the biology...) this is not very clear at the moment.	Thank you for highlighting this. We have provided further clarification by stating the following (see lines 142-143):<br><br>“This manuscript reports on the qualitative sub-study conducted within the Teen TB study.”
7.	Study design and sampling: This is done well. However please clarify why only 20 participants were selected for participation. Also, the paragraph indicates 19 participants but the table has 20- while this has been described in the abstract the same clarity is necessary in the methods.	Thank you. We have provided the following clarification. In lines 146-149:<br><br>“Twenty participants with TB disease, sampled purposively for diversity in gender, age, and drug resistance type, were invited to the qualitative study (Table 1). Nineteen participants completed all study activities; one participant was lost to attrition.”<br><br>We have also justified why only 20 participants were selected for participation as follows (see lines 150-152):<br><br>“We purposively enrolled adolescents with TB by diversity in age, gender and drug susceptibility to gain range of experiences, until reaching saturation for meaning.”<br><br>We have also added an appropriate reference:<br>Hennink MM, Kaiser BN, Marconi VC. Code Saturation Versus Meaning Saturation: How Many Interviews Are Enough? Qual Health Res. 2017;27: 591–608. doi:10.1177/1049732316665344
8.	Data collection process:<br>How was data recorded? Given the varied methods used during data collection- it was probably necessary to audio record	We have provided more detail to lines 169-172, explaining how data were recorded:

interviews and use photographs of body mapping activities- kindly indicate these. Was consent taken for recording these procedures?	"The interviews were audio recorded, and photographs taken of participatory activities, and detailed case descriptions written after each interaction with participants, which were iteratively refined with senior socio-behavioural scientists."<br><br>Lines 139-140 describe the consenting process:<br><br>"Participants were recruited during Teen TB clinical visits and consented separately for participation in the qualitative sub-study."
9. Please indicate the time taken for the interviews.	Thank you. Please see line 157-159 for details regarding the interviews:<br><br>"The qualitative data comprised 2-3 semi-structured, in-depth interviews (~30-90 minutes) with each participant over a period of about 6 months, between December 2021 to September 2022, while they were on TB treatment."
10. Where were the interviews conducted? was someone else present at the interviews or were the adolescents alone with the interviewer? Was there privacy?	Thank you. We have added the following description of where the interviews took place (see lines 159-160):<br><br>"Interviews were conducted at the clinical visits, as part of the Teen TB study. Adolescents chose who attended, controlling their privacy."
11. Data analysis: While it is understood that a deductive analysis was done, please describe the analytical approach used (thematic/content/framework).	Thank you. We have revised our description of the analytic process as follows (see lines 180-181):<br><br>"Four parameters of adolescents' experiences of TB and health service interactions, loosely based on the TB care cascade, underpinned the deductive thematic analysis"
12. Ethical considerations: Please indicate if consent for this study was obtained under the main study or if reconsenting was done.	Thank you. As per our response to comment 8 above, please see lines 149-150 which describe the consenting process:<br><br>"Participants were recruited during Teen TB clinical visits and consented separately for participation in the qualitative sub-study."

13. Additional aspects: as the analysis is deductive kindly indicate the theoretical framework/ basis for the study.	Thank you for this question. As we have indicated in our response to Reviewer 2's 3rd comment above, we used the TB care cascade as the theoretical framework. We have clarified this in lines 180-181:<br><br>"Four parameters of adolescents' experiences of TB and health service interactions, loosely based on the TB care cascade, underpinned the deductive thematic analysis."
14. Limitations (from a methods perspective): The sample size limitation does not apply to this study as it is qualitative.	Thank you, we agree. We have reworked this section as follows (see lines 343-344):<br><br>"Our study was conducted at a single site, limiting transferability."
15. It would be good to also describe the limitations/ methodological issues in terms of trustworthiness, i.e., transferability, credibility, dependability, and confirmability.	We appreciate you raising this important point about rigour in qualitative research. We agree that demonstrating credibility, dependability, and transferability is essential, and we believe our detailed description of the data collection, processing, and analysis steps already addresses this question. To clarify, we ensured credibility through the longitudinal collection of multiple data points. Dependability was maintained through regular debriefings among the research team and the iterative refinement of case descriptions, which fostered reflexivity and peer scrutiny. Finally, we established transferability through purposive sampling and by continuing data collection until saturation was reached. However, as indicated above, we acknowledge collecting data from a single-site may limit the transferability of our findings.
16. An additional note on reflexivity will be helpful.	Thank you, we have addressed this question as follows (see lines 165-167):<br><br>"We believe the fact that both research assistants were young, well-educated women, who spoke participants' mother tongue and shared their ethnic background, likely facilitated building rapport with participants and contributed to their willingness to share their experiences."

VERSION 2 - REVIEW

**Reviewer**	**1**
**Name**	**Rosnani, Zakaria**
**Affiliation**	**University Sains Malaysia - Health Campus, Department of Family Medicine**
**Date**	**19-Feb-2025**
**COI**	

Knowledge and attitude on TB among adolescents' in this community seem to be poor. Intervention module/program need to address this issue as well.

**Reviewer**	**2**
**Name**	**Lisum, Kristina**
**Affiliation**	**STIK Sint Carolus**
**Date**	**11-Mar-2025**
**COI**	

Abstract:

- the objective was not clearly stated

- how you collect the data and analized, using content analysis or??, using NVIVo, since this research is qualitative.

What is the theme that comes up from this research?

**Reviewer**	**3**
**Name**	**Rodrigues, Rashmi**
**Affiliation**	**St Johns Medical College, Community Health**
**Date**	**22-Mar-2025**
**COI**	

Please consider shortening some of the quotes - if possible.

VERSION 2 - AUTHOR RESPONSE

Reviewer 2's Comments:
In the table below, we respond to the Dr. Kristina Lisum's comments (left column) and indicate the specific changes we made to the manuscript (right column).

Comment	Response
1. Abstract:<br>- the objective was not clearly stated<br>- how you collect the data and analized, using content analysis or??, using NVIVo, since this research is qualitative.<br>- what is the theme that comes up from this research?	Thank you. We have added the following sentences to clarify the objective, and the data analysis process.<br>*We aimed to understand adolescents' experiences of TB health services in the Western Cape, South Africa.*<br><br>And,<br><br>*Fifty-two interviews were conducted and thematically analysed using a case descriptive process for experiences across the TB care cascade.*

Reviewer 3's Comments:
In the table below, we respond to the Dr. Rashmi Rodrigues' comments (left column) and indicate the specific changes we made to the manuscript (right column).

Comment	Response
1. Please consider shortening some of the quotes - if possible.	Thank you. We have shortened as many quotes as we could.
